# Territorial exceedance of probabilistic seismic hazard from ShakeMap data

**DOI:** 10.1038/s41598-024-55415-9

**Published:** 2024-02-28

**Authors:** Pasquale Cito, Antonio Vitale, Iunio Iervolino

**Affiliations:** 1 Dipartimento di Strutture Per l’Ingegneria e l’Architettura, Università degli Studi di Napoli Federico II, Via Claudio 21, 80125 Naples, Italy; 2 IUSS Scuola Universitaria Superiore di Pavia, Piazza della Vittoria 15, 27100 Pavia, Italy

**Keywords:** Civil engineering, Natural hazards, Seismology

## Abstract

Current seismic structural design makes use of a ground motion intensity that has a certain probability of being exceeded at a site of interest in a time interval or, equivalently, exceedance return period. The design intensities with the same return period are often collected in the form of maps deriving from probabilistic seismic hazard analysis (PSHA) for each of the sites of interest. Probability theory underlying PSHA dictates that, in any time interval, design intensities are expected to be exceeded in a fraction of sites that depends on the return period the map refers to. In the case of Italy, three different nationwide PSHA studies can be currently considered of relevance. In the study, the estimated areal fraction of the Italian territory exposed to exceedance of the design intensity from 2008 to 2019 was quantified for the three hazard models, based on ShakeMap data for instrumental earthquakes. In addition, the same fraction was calculated considering a sparse catalog of inferred ShakeMap for historical earthquakes that occurred over almost 1000 years. It was found that, despite the apparent differences in the hazard models, the estimated fraction of territory exposed to exceedance is comparable for all the considered hazard maps.

## Introduction

In Italy, as well as in most countries worldwide, structural design cannot neglect the possible occurrence of earthquakes at the construction site. The intensity of the seismic action to be considered in design depends on where the site is located and the target structural performance. In the framework of performance-based earthquake engineering^1^, as received by current building codes such as Eurocode 8^2^, this results in design seismic actions corresponding to the ground motion intensity measure ($IM$) value that has a certain probability of being exceeded in a given time interval or, equivalently, exceedance return period (${T}_{r}$), according to the results of the probabilistic seismic hazard analysis^3,4^ (PSHA) for the site of interest.

Several authoritative PSHA studies have been developed specifically for Italy, or at a continental scale including Italy, in the past years^5–10^. The one of Stucchi et al.^7^, MPS04 hereafter, is the PSHA officially used for seismic design of structures in the country^11^. Meletti et al.^9^ recently developed a new PSHA, indicated as MPS19, intended to replace MPS04, but not yet acknowledged by the building code^12^. The PSHA from Danciu et al.^10^, named ESHM20, has been developed for Europe and it is foreseen as an annex to the new generation of Eurocode 8 and therefore is also of relevance for Italy. MPS04, MPS19 and ESHM20 rely on different models for each of the main PSHA components, and therefore the results they provide for an area of interest show differences discussed by some dedicated studies^13^.

The evaluation of PSHA results based on ground motion observations is the subject of scientific and sometimes also public debate^14^. In this context, it has been demonstrated that the exceedance of design $IM$ values from PSHA is to be expected, especially in the epicentral area of moderate-to-large magnitude earthquakes^15^, and that this does not necessarily mean that PSHA underestimates the actual seismic hazard, unless it is proven that such exceedances are significantly more frequent than what stipulated by the hazard assessment. At a single site, formal validations of this kind are seldom feasible due to the rarity of strong ground motion records that would require several thousands of years^16,17^, whilst an alternative is to pool data from multiple sites^18,19^. Some studies also theorize the use of regional estimates of ground shaking, due to earthquakes that have occurred in a time interval, to compare with what is expected from PSHA results^20,21^.

In Italy, ground motion at a large scale is provided in near-real-time by the *Istituto Nazionale di Geofisica e Vulcanologia* (INGV), which currently implements ShakeMap (v4.0) software^22^. For any earthquake (with magnitude equal to or larger than 3.0) the Italian ShakeMap implementation provides territorial assessment of $IM$s defined as peak ground acceleration or $PGA$, pseudo-spectral acceleration associated with a vibration period equal to 0.3 s, 1.0 s and 3.0 s, indicated as $Sa\left(0.3s\right)$, $Sa\left(1.0s\right)$ and $Sa\left(3.0s\right)$, respectively, and macroseismic intensity in terms of the *Mercalli-Cancani-Sieberg* scale^23^. ShakeMap provides the shaking at the nodes of a grid, with 1 km spacing, in the impacted area, also where there are no observations available. This is done assuming that the logarithms of the $IM$ of interest at all the sites, in the considered area, are represented by a Gaussian random field (GRF) conditional to magnitude of the earthquake, its location, and the $IM$ values at the sites with recording stations^24^. Consequently, for each point of the grid, ShakeMap also quantifies the uncertainty associated with the $IM$ at that point. ShakeMap data, which pertains to earthquakes from 2008 onwards, are publicly available through an online repository (see “Data availability”).

Results of classical PSHA are often represented in terms of hazard maps, each of which refers to a given ${T}_{r}$. Each map collects the $IM$ values that, at each site (taken individually) have the same probability of being exceeded ($p$) at least once in the $\left(t,t+\Delta t\right)$ time interval, and this probability is equal to $p=1-{e}^{-\Delta t/{T}_{r}}$. This means that, at any site, the exceedance of the $IM$ values from the map occurs in $\left(t,t+\Delta t\right)$ with probability $p$ and does not occur with probability $1-p$. In other words, exceedance in $\left(t,t+\Delta t\right)$ is a Bernoulli random variable whose mean is $p$. It is interesting, for the purposes of this study, to note that this simple result enables a sort of *trading of time for space*. In fact, the expected value of the sites experiencing at least one exceedance is the mean of the sum of the Bernoulli random variables at all sites represented in the hazard map, say they are $N$ in number. Because the mean of the sum of $N$ random variables is always (without any other needed assumption) the sum of the mean of each random variable^25^, which in the selected case is always $p$ by definition of hazard map, then such expected value is $N\cdot p$. Thus, the expected fraction of sites where at least one exceedance is observed in $\left(t,t+\Delta t\right)$ is $N\cdot p/N=p$. For example, it is expected that in 50 years 10% of the sites experience at least one exceedance of the $IM$ values from the hazard map referring to ${T}_{r}=475 {\text{ years}}$, because $1-{e}^{-50/475}=0.1$.

To assess how the estimated exceedance area in actual earthquake events compares with what is expected from PSHA is the aim of this study. ShakeMap data for, instrumentally recorded, earthquakes that occurred in Italy from 2008 to 2019 is compared to some of the maps of most relevant engineering interest, from MPS04, MPS19, and ESHM20, considering return periods between 50 and 2475 years. In addition, a recently developed large database of ShakeMap inferred from macroseismic intensity data, for a sparse set of so-called historical earthquakes (i.e., that occurred in pre-instrumental era) spanning almost 1000 years^26^, is also considered.

A similar concept as the one put forward in this study has been recently applied in other countries^27^, and although it is not intended herein as a formal testing of any of the PSHA models, which would require one to compute (for each of the PSHA studies) the distribution of the random variable representing the exceedance area in 12 years, it still enables a simple comparison of hazard maps against available data.

## Results and discussions

### Seismic hazard maps for Italy

The main input components needed for PSHA, that is a hazard model, are: one or a set of seismological source models, which includes a probabilistic characterization of future location and magnitude of earthquakes, typically built starting from a catalog collecting information about past earthquakes (even if earthquakes are clustered in space and time, only the largest magnitude events in each cluster, the so-called mainshocks, are considered in classical PSHA), and at least one ground motion model (GMM)^28^.

MPS04 relies on 36 seismic source zones^29^. Even if it features a logic tree with 16 branches, only the models of branch named 921 are considered herein. In branch 921, which is claimed to be the one with the results that are closest to those representing the median of the full logic tree^7,30^, seismicity is defined, for each source zone, in terms of the so-called *activity rates*, that is, annual rates of earthquakes associated with surface-wave magnitude (M_s_) bins that are 0.3 magnitude units wide^31^. The lowest magnitude bin is centered at M_s_ 4.3, that is, minimum magnitude of earthquakes is 4.15, for all zones (with the exception of the Etna’s volcanic area in eastern Sicily, being the minimum magnitude equal to M_s_ 3.55), whereas the largest magnitude bin can be as high as 7.45 (i.e., the largest magnitude bin is centered at M_s_ 7.3), depending on the zone. The GMM is that of Ambraseys et al.^32^. The predominant style-of-faulting of the sources is also accounted for in the PSHA via the correction factors proposed by Bommer et al.^33^.

MPS19 is based on 94 source models, each of which is assigned a weight based on an experts’ elicitation procedure^34^. These models are combined with GMMs via a logic tree featuring about 600 branches overall. However, a relatively easy-to-implement weighted average grid-seismicity source model, covering Italy and the surrounding areas via 11,000 point-like seismic sources, was also derived from the ensemble of the logic tree, by the same working group that developed MPS19^35^. For each point source, seismicity is defined in terms of activity rates associated with 46 moment magnitude (M_w_) bins with width equal to 0.1 magnitude units. The minimum magnitude bin is centered at M_w_ 4.5, so that the minimum magnitude of earthquakes is 4.45, while the largest magnitude is M_w_ 9.05 in about 85% of the country and M_w_ 8.35 in the remaining areas (however, the activity rates associated with such magnitudes are small if not negligible). A probabilistic distribution of the style-of-faulting is also defined for each point source. This seismicity model, coupled with the GMM of Bindi et al.^36^, which is deemed as the best performing one for Italy^37^, represents the second PSHA considered.

For Italy, ESHM20 adopts a logic tree consisting of two main branches, one for the area source models and the other for active faults coupled with background seismicity. Minimum magnitude is M_w_ 4.5, whereas the largest value can be as high as M_w_ 8.4, depending on the seismic source. The GMM adopts the so-called *backbone* approach, in which region-dependent adjustment factors are applied to the GMM of Kotha et al.^38^. The GMM accounts for epistemic uncertainty by means of a 9 branches logic tree^39^. The ESHM20 implementation herein only considers the two branches for the source models and one branch for the GMM, that is the one with the largest weight. This set of models is claimed by the ESHM20 developers to provide PSHA results that better approximate those corresponding to the mean of the ESHM20 logic tree.

**Figure 1:**
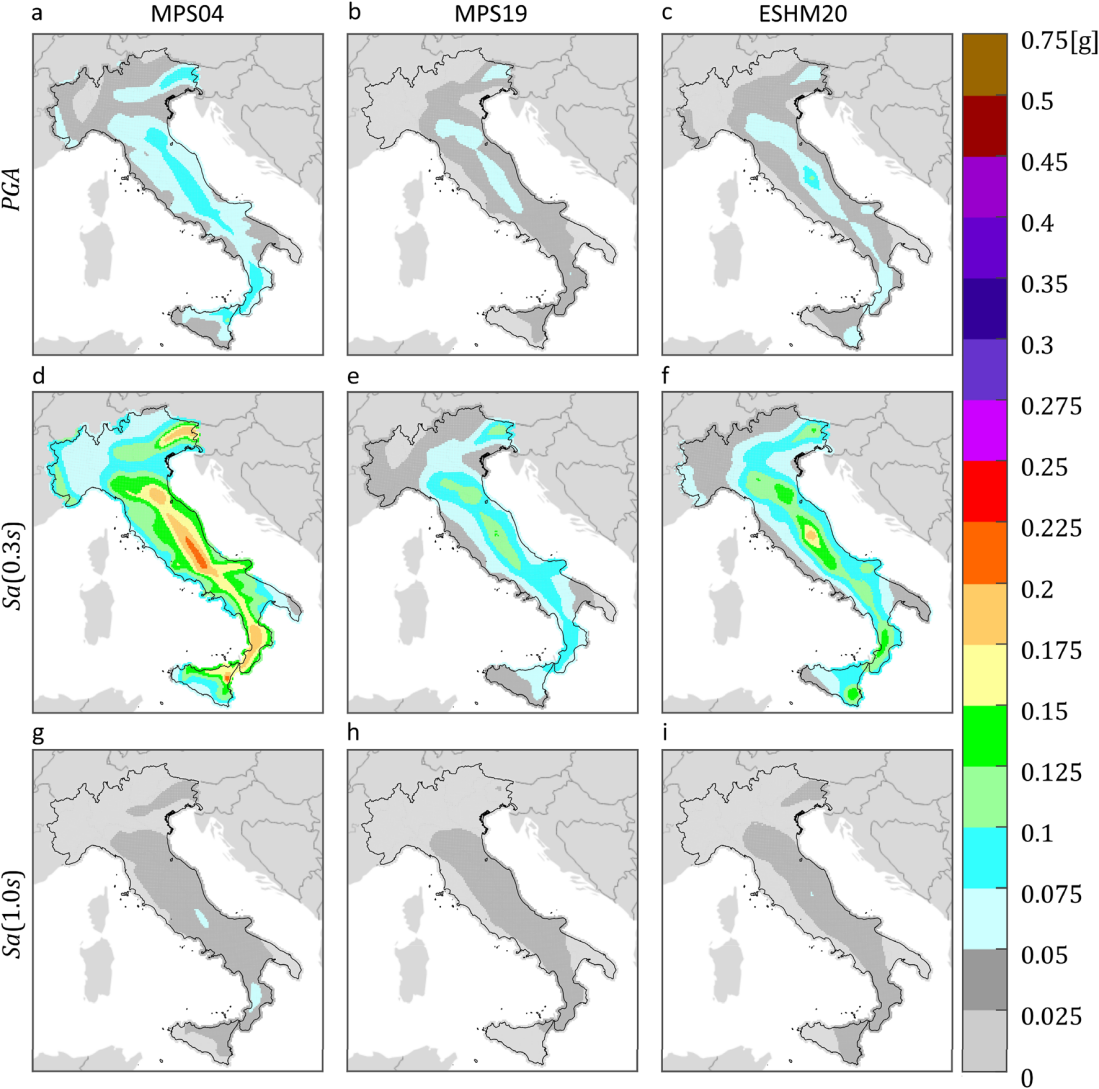
${T}_{r}=50 {\text{ years}}$ hazard maps for three intensity measures, on rock, according to three PSHA models. The maps in the same column refer to the same PSHA study: (**a,d,g**) are from MPS04; (**b,e,h**) are from MPS19; (**c,f,i**) are from ESHM20. The maps in the same row refer to the same $IM$: (**a–c**) are $PGA$; (**d–f**) are $Sa\left(0.3s\right)$; (**g–i**) are $Sa\left(1.0s\right)$. The maps were generated using the Matlab mapping toolbox version 4.10 (https://it.mathworks.com/products/mapping.html).

**Figure 2:**
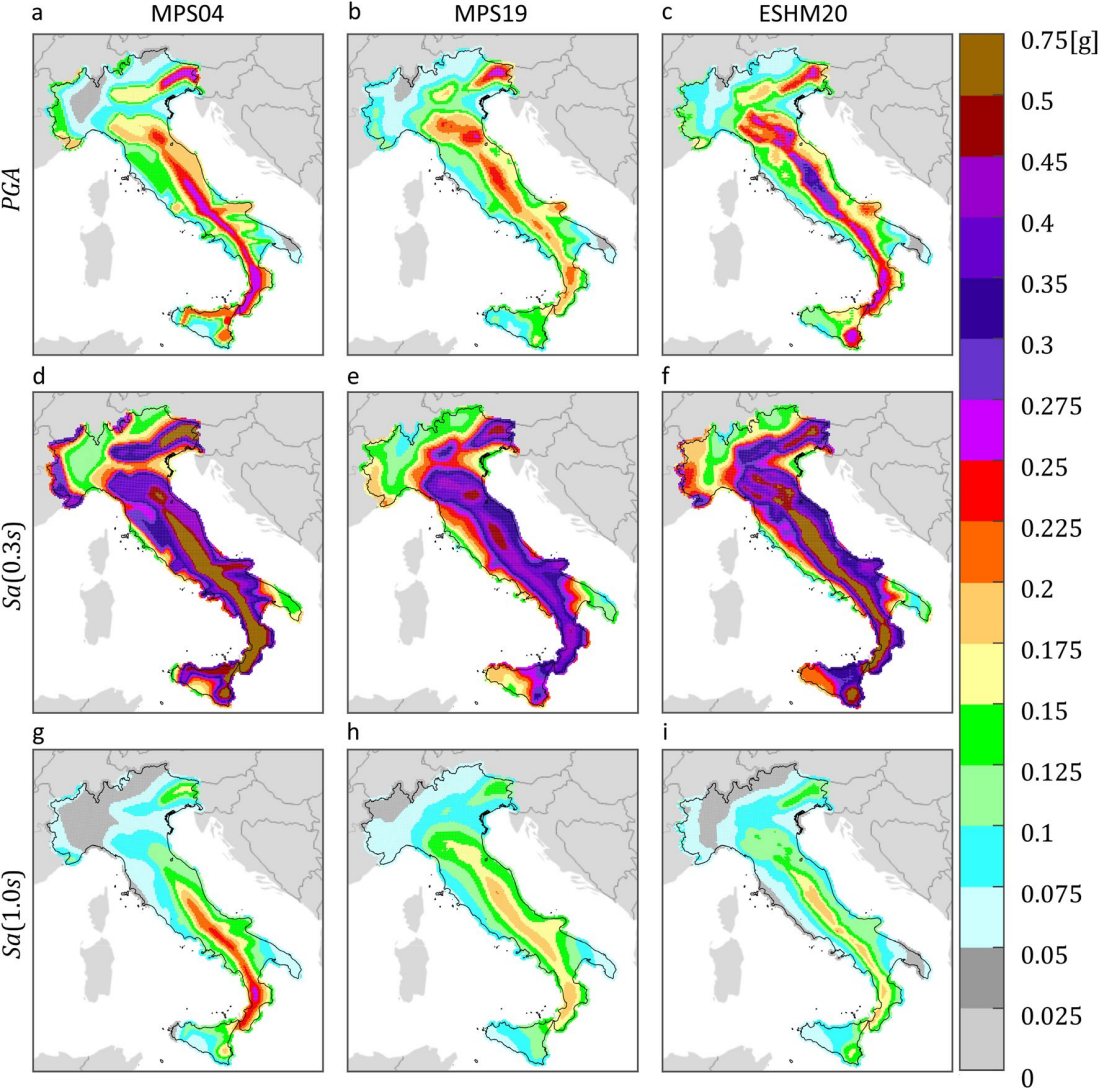
${T}_{r}=475 {\text{ years}}$ hazard maps for three intensity measures, on rock, according to three PSHA models. The maps in the same column refer to the same PSHA study: (**a,d,g**) are from MPS04; (**b,e,h**) are from MPS19; (**c,f,i**) are from ESHM20. The maps in the same row refer to the same $IM$: (**a–c**) are $PGA$; (**d–f**) are $Sa\left(0.3s\right)$; (**g–i**) are $Sa\left(1.0s\right)$. The maps were generated using the Matlab mapping toolbox version 4.10 (https://it.mathworks.com/products/mapping.html).

Hazard maps on reference rock site conditions (A site class according to Eurocode 8) were derived for the purposes of this study, 12 for each PSHA study. The maps refer to three $IMs$, that are $PGA$, $Sa\left(0.3s\right)$ and $Sa\left(1.0s\right)$, and four return periods, 50, 475, 975, and 2475 years. The maps were computed, via the REASSESS ^40^ software (see “Code availability”), on a grid of about 10,000 sites which exclude the Sardinia Island, as MPS04 does not provide hazard results for it. Because the GMMs at the basis of MPS04, MPS19 and ESHM20 treat the two horizontal components of ground motion in different manners, results from MPS19 and ESHM20 were adjusted to be comparable with MPS04 ones, which are in terms of largest component, according to the model of Beyer and Bommer^41,42^. Figures 1 and 2 show the hazard maps for ${T}_{r}=50 {\text{ years}}$ and ${T}_{r}=475 {\text{ years}}$, respectively, whereas maps for ${T}_{r}=975 {\text{ years}}$ and ${T}_{r}=2475 {\text{ years}}$ are not shown, but they are provided in the supplementary material (see “Data availability”).

The figures show that, for the same return period and $IM$, seismic hazard can apparently vary in some areas of the country, depending on the PSHA model the hazard map refers to. Looking at the maps with ${T}_{r}=50 {\text{ years}}$, it appears that MPS04 tends to provide the largest values, for any $IM$, especially in the northeast and along the Apennines. This is also found in the case of  ${T}_{r}=475 {\text{ years}}$, when the $IM$ is in terms of $Sa\left(1.0s\right)$. Considering $PGA$, it seems that ESHM20 provides the largest seismic hazard. For example, in central Italy, which is one of the most hazardous regions in the country according to all PSHA studies, the relative difference with the $PGA$ values from MPS04 and MPS19 is up to about 60% and 70%, respectively. Looking at $Sa\left(0.3s\right)$, it is found that MPS04 and ESHM20 are in relatively good accordance, while MPS19 provides the lowest values.

Finally, it is remarked that to consider only one branch per hazard model in lieu of the logic tree, or to use simplified source models giving PSHA results that well approximate the mean (or median) of the whole logic tree, is for reproducibility and to control the results. This does not enable one to explore the effect of so-called epistemic uncertainty in the context of exceedance area, yet it is consistent with evaluation of the main PSHA results which are represented by the weighted average (or the median) of the logic tree branches’ results.

### Exceedance area in continuously monitored 12 years

The estimated extent of the regions where the largest ground motion intensity from ShakeMap, due to instrumentally-recorded earthquakes that occurred from 2008 to 2019, exceeds the thresholds from the hazard maps, was quantified for each of the possible triplets of PSHA model, $IM$ and return period, that is, 36 cases. Given the return period, the expected value of the exceedance area depends only on the width of the time interval of interest. Considering the definition of the probability of exceedance $\left(p\right)$ given above, the expected area experiencing at least one exceedance, in 12 years, of the $IM$ values from the hazard map with ${T}_{r}=50 {\text{ years}}$ is 21.3% of map. This percentage reduces to 2.5%, 1.2%, and 0.5% for ${T}_{r}=475 {\text{ years}}$, ${T}_{r}=975 {\text{ years}}$ and ${T}_{r}=2475 {\text{ years}}$, respectively.

Even if insights on ShakeMap data are given in the Methods section, it is worthwhile anticipating here that, for each $IM$, the sought exceedance areas stem from the comparison between the largest values in the country from ShakeMap and the hazard maps for different hazard models and return periods. To this aim, the maximum among the median ShakeMap intensities for the earthquakes recognized as mainshocks and with magnitude larger than the minimum considered by the considered hazard models, were taken at each site. Then, the exceedance areas were divided by the area covered by hazard maps to get the fractions of the country exposed to exceedance.

The resulting fractional areas are given in Table 1, together with the expected values for each return period. In the cases of ${T}_{r}=50 {\text{ years}}$ and ${T}_{r}=475 {\text{ years}}$, the exceedance area in 12 years, from ShakeMap, is comparable across $IMs$ and hazard models. This holds, in general terms, also for ${T}_{r}=975 {\text{ years}}$. For ${T}_{r}=2475 {\text{ years}}$, the exceedance area is equal to or close to zero, because these hazard thresholds are relatively hard to be exceeded in such a short period.

**Table 1:** Estimated fraction of Italy exposed to exceedance, due to mainshocks that occurred from 2008 to 2019, of the PSHA intensity thresholds in terms of three $IMs$ with four site-specific return periods, according to three PSHA studies.

	Expect. fractional area (%)	Empirical for MPS04			Empirical for MPS19			Empirical for ESHM20		
		$PGA$ (%)	$Sa\left(0.3s\right)$ (%)	$Sa\left(1.0s\right)$ (%)	$PGA$ (%)	$Sa\left(0.3s\right)$ (%)	$Sa\left(1.0s\right)$ (%)	$PGA$ (%)	$Sa\left(0.3s\right)$ (%)	$Sa\left(1.0s\right)$ (%)
${T}_{r}=50 {\text{ years}}$	21.3	4.42	3.40	5.11	6.00	5.40	4.36	6.29	5.67	6.33
${T}_{r}=475 {\text{ years}}$	2.5	0.90	0.48	0.79	0.39	0.32	0.29	0.34	0.33	0.64
${T}_{r}=975 {\text{ years}}$	1.2	0.31	0.08	0.45	0.12	0.10	0.09	0.05	0.09	0.23
${T}_{r}=2475 {\text{ years}}$	0.5	0.01	0	0.09	0.01	0	0	0.01	0	0.08

It also appears that, for each return period, the estimated fraction of the territory exposed to exceedance in the available 12 years is one order of magnitude lower (or slightly less) than the expected value according to all PSHA studies.

**Figure 3:**
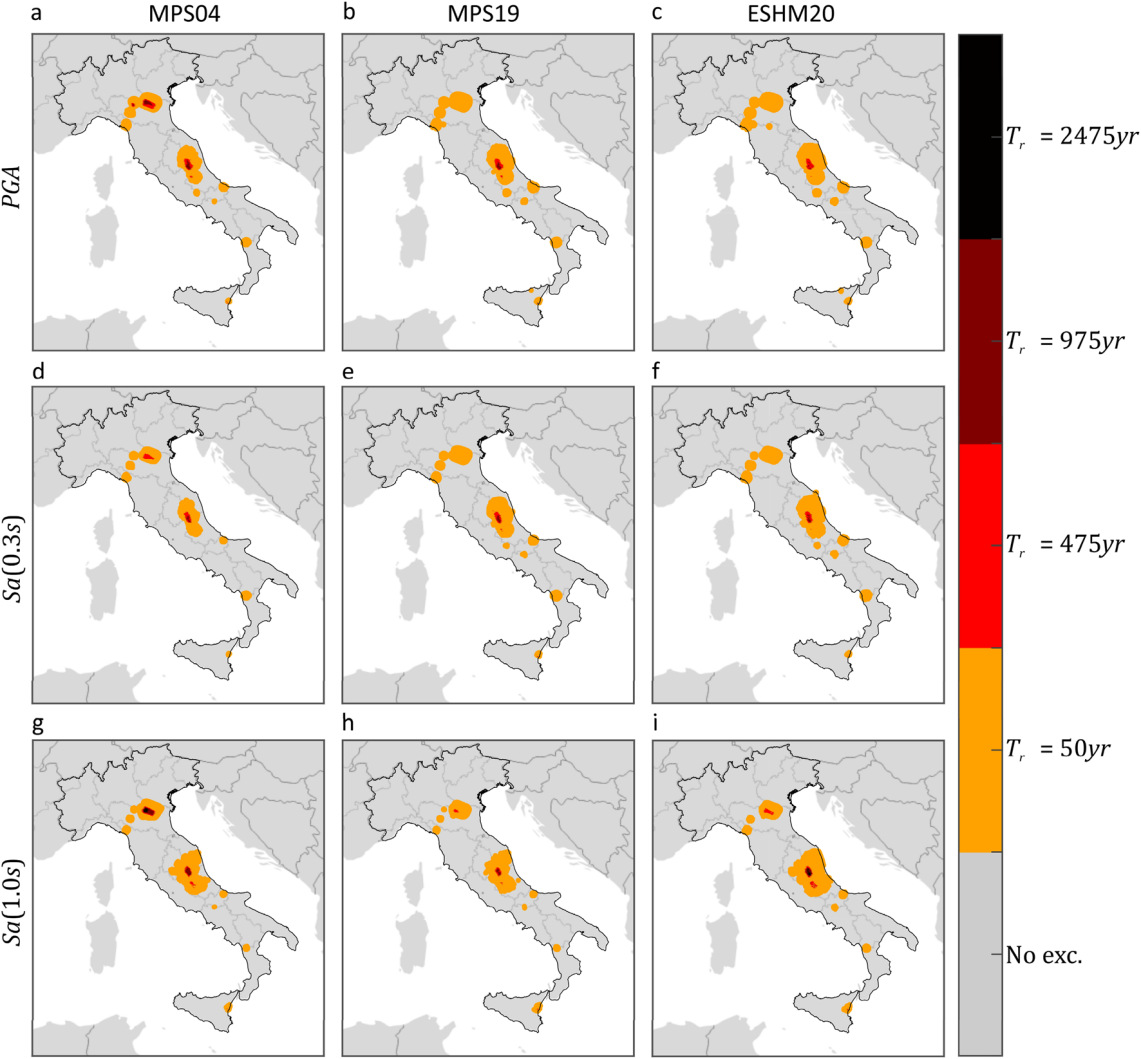
Areas subjected to at least one exceedance of the PSHA intensity thresholds, in terms of three $IMs$ corresponding to three PSHA studies, due to mainshocks that occurred from 2008 to 2019. The maps were developed by comparing the hazard maps with the map of the largest intensities according to ShakeMap. Markers’ color indicates the largest return period for which at least one exceedance is estimated. The maps in the same column refer to the same PSHA study: (**a,d,g**) are from MPS04; (**b,e,h**) are from MPS19; (**c,f,i**) are from ESHM20. The maps in the same row refer to the same $IM$: (**a–c**) are $PGA$; (**d–f**) are $Sa\left(0.3s\right)$; (**g–i**) are $Sa\left(1.0s\right)$. The maps were generated using the Matlab mapping toolbox version 4.10 (https://it.mathworks.com/products/mapping.html).

Figure 3 shows, for each PSHA model and $IM$, where ShakeMap indicates at least one exceedance in 12 years. The exceedance areas of the $IM$ values with 50 years return period are plotted in orange, while red, amaranth, and black are used for the exceedance of the $IM$ values with return periods equal to 475, 975, and 2475 years, respectively. Gray indicates sites where no exceedance was expected by ShakeMap (see “Methods”). These figures also confirm that the differences between the PSHA models seem to have a limited effect on the area where at least one exceedance has possibly occurred from 2008 to 2019.

These results show that, based on the available regional shaking estimates for Italy, the estimated fraction of the country exposed to at least one exceedance from 2008 to 2019 is comparable for all the hazard models, despite their apparent differences.

**Figure 4:**
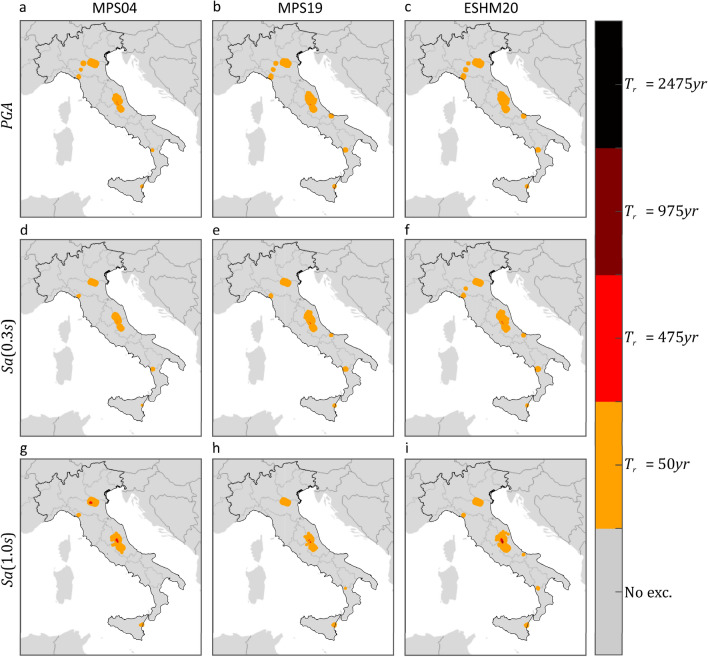
Areas subjected to at least one exceedance of the PSHA intensity thresholds, in terms of three $IMs$ corresponding to three PSHA studies, due to mainshocks that occurred from 2008 to 2019. The maps were developed by comparing the hazard maps with the map of the largest intensities, according to ShakeMap, after having subtracted, at each site, the value representative of the uncertainty. Markers’ color denotes the largest return period for which at least one exceedance is estimated. The maps in the same column refer to the same PSHA study: (**a,d,g**) are from MPS04; (**b,e,h**) are from MPS19; (**c,f,i**) are from ESHM20. The maps in the same row refer to the same $IM$: (**a–c**) are $PGA$; (**d–f**) are $Sa\left(0.3s\right)$; (**g–i**) are $Sa\left(1.0s\right)$. The maps were generated using the Matlab mapping toolbox version 4.10 (https://it.mathworks.com/products/mapping.html).

**Figure 5:**
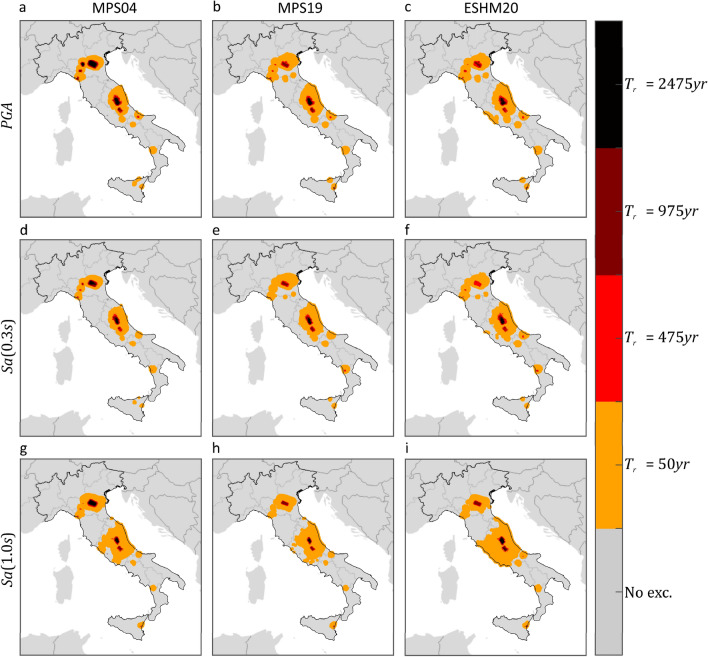
Areas subjected to at least one exceedance of the PSHA intensity thresholds, in terms of three $IMs$ corresponding to three hazard models, due to mainshocks that occurred from 2008 to 2019. The maps were developed by comparing the hazard maps with the map of the largest intensities, according to ShakeMap, after having added, at each site, the value representative of the uncertainty. Markers’ color denotes the largest return period for which at least one exceedance is estimated. The maps in the same column refer to the same PSHA study: (**a,d,g**) are from MPS04; (**b,e,h**) are from MPS19; (**c,f,i**) are from ESHM20. The maps in the same row refer to the same $IM$: (**a–c**) are $PGA$; (**d–f**) are $Sa\left(0.3s\right)$; (**g–i**) are $Sa\left(1.0s\right)$. The maps were generated using the Matlab mapping toolbox version 4.10 (https://it.mathworks.com/products/mapping.html).

As mentioned, ShakeMap also provides a so-called *error* term, which aims at quantifying the uncertainty affecting the provided (median) ground motion. Because of the conditional GRF modelling entailed by ShakeMap, such a term accounts for all the uncertainties considered in inter- and intra-event residuals of ground motion models^43^. The ShakeMap error term is taken into account to have a measure of the variability of the fractional exceedance area, when the uncertainty affecting ShakeMap is considered. To do so, two additional maps were computed for each $IM$. The first is obtained taking the maximum among (i.e., enveloping them, see "Methods") the median ShakeMap intensities considered above, yet after having subtracted, at each site, one standard deviation. The second map is built by taking, at each site, the maximum ShakeMap intensity with one standard deviation being added (subtraction and addition are both carried out in logarithmic space). The comparison of these maps with the hazard maps gives a rough idea of the *one-sigma* variability of the exceedance area due to ShakeMap uncertainty. These results are given in Table 2, confirming that the exceedance area is comparable for all PSHA model, even when ShakeMap uncertainty is considered. Taking the ensemble of PSHA models and $IM$s considered in the study, the lower fractional exceedance area varies in the 1.3–2.5% range when ${T}_{r}=50 {\text{ years}}$, whereas it approaches to zero for the larger return periods. The upper exceedance fractional area gets close or even exceeds the expected value. In the case of ${T}_{r}=50 {\text{ years}}$, the largest value, obtained when the hazard map in terms of $Sa\left(1.0s\right)$ from ESHM20 is considered, is equal to 17.2%, against an expected value of 21.3%. At larger return periods, the fraction of country exposed to exceedance, when ShakeMap intensities are amplified due to uncertainty, becomes larger, even if only slightly, when the hazard intensities from the MPS04 hazard map in terms of $PGA$ are considered. Finally, Figs. 4 and 5 provide a graphical representation of such variability of the fractional exceedance area due to ShakeMap uncertainty. Once again, the patterns of the maps seem to be relatively invariant with respect to the hazard model to which the intensity thresholds correspond.

**Table 2:** Effect of ShakeMap uncertainty on the estimated fraction of the country exposed to exceedance, due to mainshocks that occurred from 2008 to 2019, of three $IMs$ with four site-specific return periods, according to three hazard models.

	Expect. fractional area (%)		Empirical for MPS04			Empirical for MPS19			Empirical for ESHM20		
			$PGA$ (%)	$Sa\left(0.3s\right)$ (%)	$Sa\left(1.0s\right)$ (%)	$PGA$ (%)	$Sa\left(0.3s\right)$ (%)	$Sa\left(1.0s\right)$ (%)	$PGA$ (%)	$Sa\left(0.3s\right)$ (%)	$Sa\left(1.0s\right)$ (%)
${T}_{r}=50 {\text{ years}}$	21.3	Low.	1.60	1.25	1.79	2.38	2.09	1.53	2.5	2.20	1.98
		Up.	9.58	8.22	12.68	13.07	12.36	11.15	13.14	12.33	17.23
${T}_{r}=475 {\text{ years}}$	2.5	Low.	0.02	0.007	0.23	0.01	0.01	0.04	0.007	0.015	0.16
		Up.	2.87	1.89	1.80	1.96	1.62	1.04	2.08	1.88	1.55
${T}_{r}=975 {\text{ years}}$	1.2	Low.	0	0	0.02	0.003	0	0	0.002	0	0.04
		Up.	1.85	1.08	1.13	0.97	0.84	0.61	0.89	0.68	0.95
${T}_{r}=2475 {\text{ years}}$	0.5	Low.	0	0	0	0	0	0	0	0	0
		Up.	0.95	0.40	0.58	0.29	0.19	0.11	0.22	0.22	0.33

What discussed so far shows that the (estimated) size of the exceedance area may differ from the expected values by an amount that can be even considered large. However, as already mentioned above, the comparison of ShakeMap data with the expected exceedance area from hazard maps is not sufficient to evaluate the performance of any of the considered PSHA models, as any formal testing would require one to compute the whole probability distribution of the exceedance area according to such PSHA models, similar to what done by the authors in other studies^19,44^.

### Exceedance area from historical earthquakes

Similar to the previous section, the fraction of the Italian territory subjected to at least one exceedance due to non-instrumental earthquakes that occurred from 1117 to 1968 was quantified for 36 combinations of PSHA models, $IM$ and return periods. The expected fractional exceedance area in the 852 years period was not quantified because the considered dataset of historical earthquakes is not complete (see "ShakeMap data for historical earthquakes").

**Figure 6:**
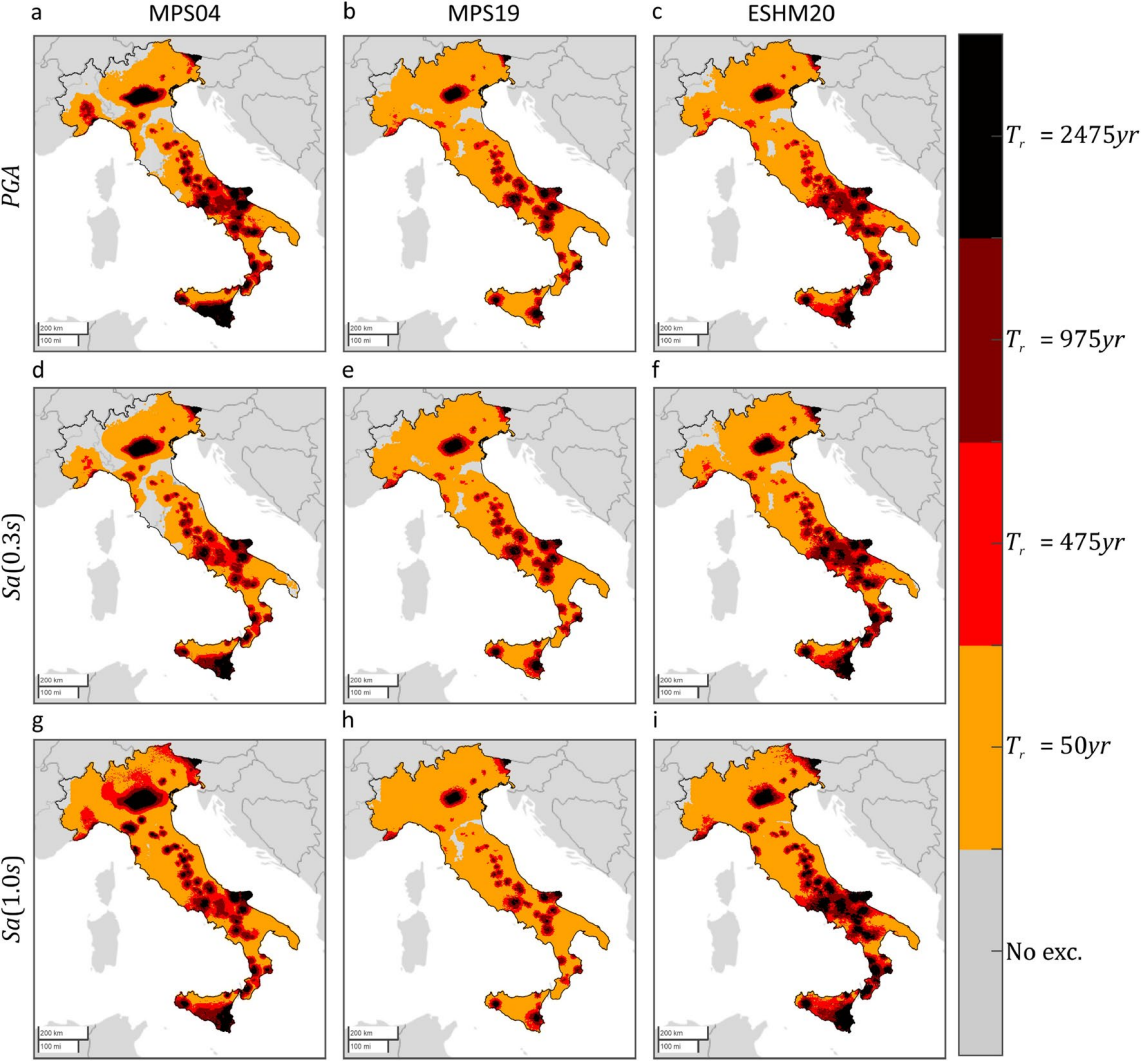
Areas subjected to at least one exceedance of the PSHA intensity thresholds, in terms of three $IMs$ corresponding to three PSHA studies, due to mainshocks (whose ShakeMap are available) that occurred from 1117 to 1968. The maps were developed by comparing the hazard maps with the map of the largest intensities according to ShakeMap. Markers’ color indicates the largest return period for which at least one exceedance is estimated. The maps in the same column refer to the same PSHA study: (**a,d,g**) are from MPS04; (**b,e,h**) are from MPS19; (**c,f,i**) are from ESHM20. The maps in the same row refer to the same $IM$: (**a–c**) are $PGA$; (**d–f**) are $Sa\left(0.3s\right)$; (**g–i**) are $Sa\left(1.0s\right)$. The maps were generated using the Matlab mapping toolbox version 4.10 (https://it.mathworks.com/products/mapping.html).

For each $IM$, return period and PSHA model, the sought exceedance area was obtained from the comparison of the hazard map with the map collecting, at each site, the maximum among the median ShakeMap intensities for 71 (out of a total of 76) historical earthquakes, with moment magnitude larger than M_w_ 6.0, that are recognized as mainshocks^26^. Such areas are given in Table 3, where it can be observed that, given return period, $IM$ and hazard model, the exceedance area increases significantly with respect to that found due to instrumental mainshocks from 2008 to 2019, as expected, given the comparatively larger magnitude. However, given $IM$ and return period, the effect of the hazard model on the value of such area is still limited, as observed for the dataset of instrumental events. This is also confirmed by Fig. 6 graphically, where the areas experiencing at least one exceedance are shown.

**Table 3:** Estimated fraction of Italy exposed to exceedance, due to historical mainshocks, of the PSHA intensity thresholds in terms of three $IMs$ with four site-specific return periods, according to three PSHA studies.

Empirical exceedance area	MPS04			MPS19			ESHM20		
	$PGA$ (%)	$Sa\left(0.3s\right)$ (%)	$Sa\left(1.0s\right)$ (%)	$PGA$ (%)	$Sa\left(0.3s\right)$ (%)	$Sa\left(1.0s\right)$ (%)	$PGA$ (%)	$Sa\left(0.3s\right)$ (%)	$Sa\left(1.0s\right)$ (%)
${T}_{r}=50 {\text{ years}}$	78.4	76.6	97.3	93.4	93.4	96.6	92.5	91.9	98.2
${T}_{r}=475 {\text{ years}}$	33.3	29.0	42.4	20.5	22.7	17.3	31.3	35.1	41.7
${T}_{r}=975 {\text{ years}}$	21.5	16.7	21.8	10.3	11.3	7.6	15.9	20.3	25.8
${T}_{r}=2475 {\text{ years}}$	9.5	6.0	8.1	3.0	3.1	2.0	4.9	7.3	11.5

The results for historical earthquakes confirm the conclusion about the comparability of the hazard maps from the considered hazard models with respect to the areas experiencing at least one exceedance.

## Methods

### ShakeMap data for instrumental events

The ShakeMap service of INGV estimates the intensity of ground shaking in the earthquake impacted area via a GRF based on GMMs and correlation models for GMM residuals. These models drive the results of ShakeMap and those used in Italy were selected based on a ranking procedure discussed in Michelini et al.^23^.

**Figure 7:**
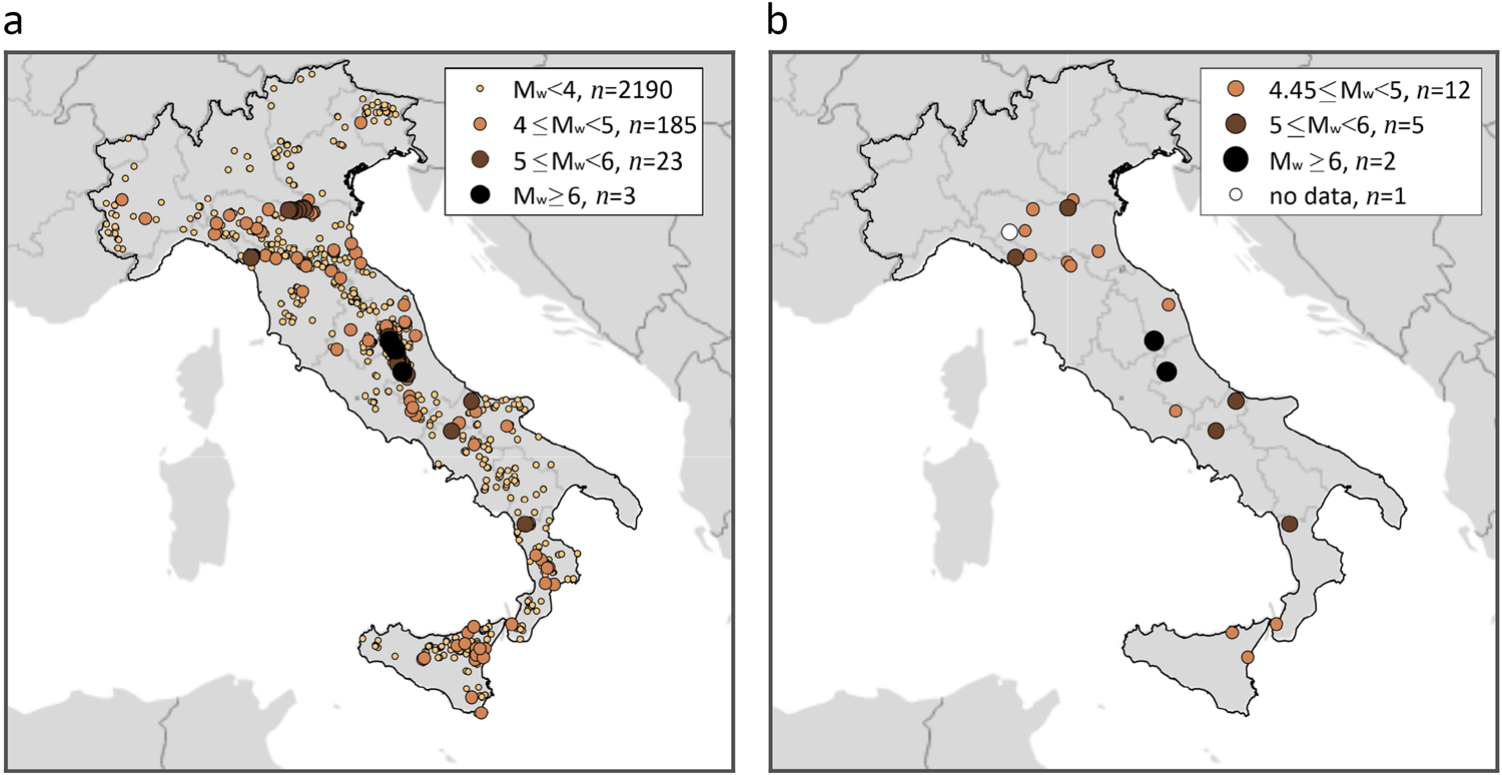
(**a**) Magnitude and location of all earthquakes processed by ShakeMap from 2008 to 2019. (**b**) Magnitude and location of the M_w_ 4.45+ events that, according to the declustering analysis of CPTI15, are identified as mainshocks and considered in this study (non-white dots). The maps were generated using the Matlab mapping toolbox version 4.10 (https://it.mathworks.com/products/mapping.html).

The ShakeMap implementation in Italy provides, for the period time from 2008 to 2019, data for 2401 earthquakes occurring in the mainland Italy and Sicily, whose epicenters are shown in Fig. 7a. The markers’ size and color are a function of the event moment magnitude, which is between M_w_ 2.9 and M_w_ 6.5 overall. In the figure, the number of earthquakes $\left(n\right)$ for each magnitude interval is also given.

Three (extensively damaging) earthquakes with magnitude equal to or larger than 6.0 occurred in the considered period: L’Aquila 2009 (M_w_ 6.1)^45^, while the remaining two, that is, Accumoli 2016 (M_w_ 6.0), and Norcia 2016 (M_w_ 6.5), belong to the 2016–2017 central Italy seismic sequence^46^. However, to be consistent with the PSHA studies considered, the dataset for the evaluation of the exceedance area should only collect ShakeMap from the mainshocks. Herein, the mainshocks from the *Catalogo Parametrico dei Terremoti Italiani* or CPTI15 were considered^47^. (In principle, the catalog should be the same as the one at the basis of the hazard model, if available.) Note that the version of CPTI15 currently available is not declustered. However, a declustered version of the catalog, which includes mainshocks up to 2019, was obtained by INGV (see “Data availability”) and this is the reason why ShakeMap data pertaining to earthquakes after 2019 was not considered in the study. In addition, mainshocks with moment magnitude lower than the minimum assumed by the considered hazard models, were neglected (see "Seismic hazard maps for Italy" section). Thus, the number of remaining earthquakes considered in this study reduces from 2401 to 19 in the case of MPS19 or ESHM20, and 16 for MPS04.

Figure 7b represents moment magnitude and location of the earthquakes in the beginning and those for which ShakeMap are finally considered. It appears that the declustering of the catalog ends in not considering several earthquakes with magnitude below 5.0, as they are not identified as mainshocks. For instance, several earthquakes with moment magnitude between M_w_ 5.0 and M_w_ 6.0, that occurred in the 2016–2017 central Italy sequence, were removed. It should also be noted that ShakeMap data miss one mainshock with magnitude M_w_ 5.05 according to CPTI15; it is indicated as white circle in the figure, and it does not contribute to the results discussed above.

### ShakeMap data for historical earthquakes

**Figure 8:**
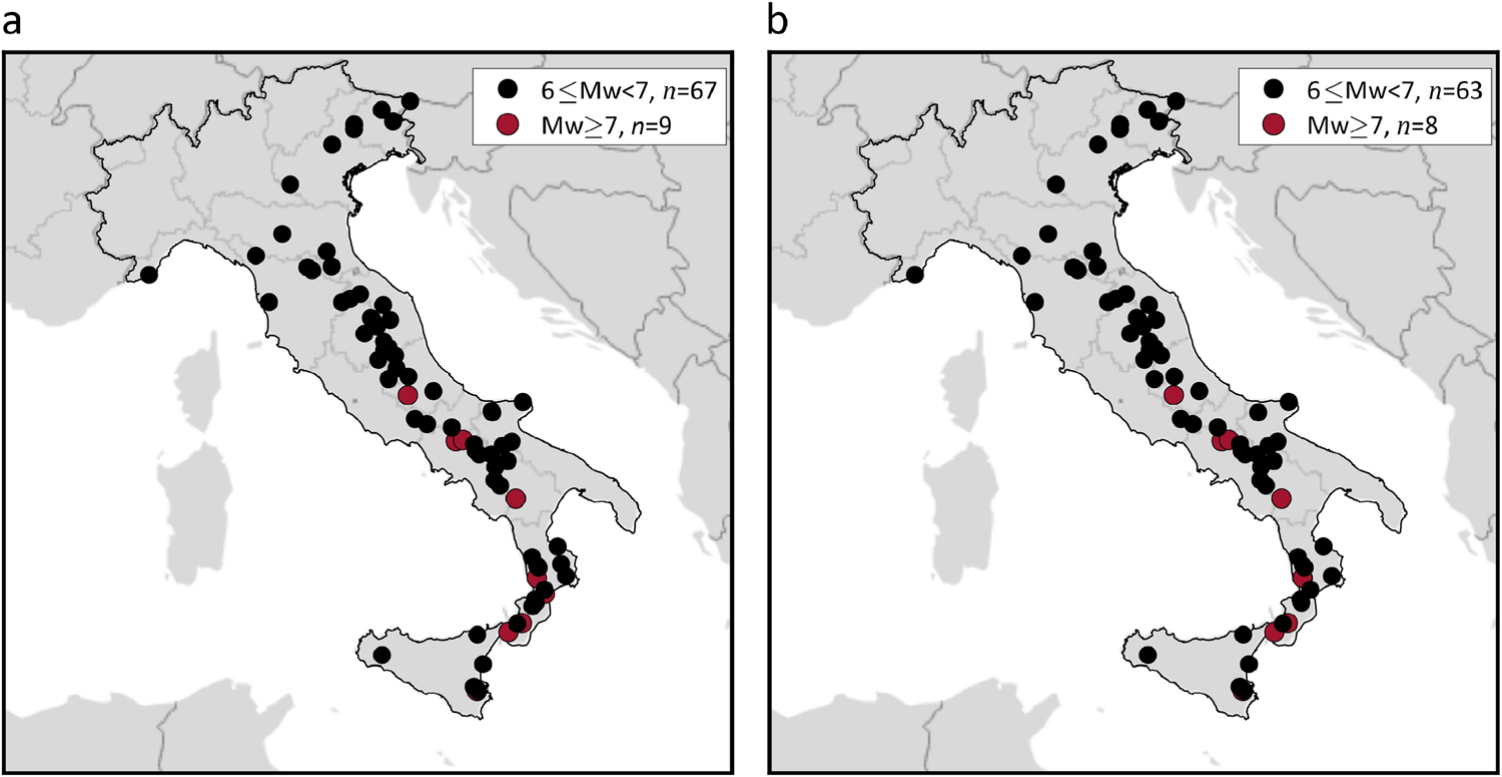
(**a**) Magnitude and location of all historical earthquakes, whose ShakaMap are available, from 1117 to 1968. (**b**) Magnitude and location of the M_w_ 6.0+ events that, according to the declustering analysis of CPTI15, are identified as mainshocks and considered in this study. The maps were generated using the Matlab mapping toolbox version 4.10 (https://it.mathworks.com/products/mapping.html).

Italy has one of the longest records of historical earthquakes in the world, that is, earthquakes that occurred in pre-instrumental era for which source and effect information can be inferred from other sources. For a selection of 76 earthquakes, with moment magnitude between M_w_ 6.0 and M_w_ 7.3 that occurred across Italy from 1117 to 1968, ShakeMap have been recently made available based on macroseismic intensity data^26^. The epicenters of these events are represented in Fig. 8a, where black dots denote event with magnitude between M_w_ 6.0 and M_w_ 7.0, while amaranth ones represent epicenters of those with magnitude larger than M_w_ 7.0. Among the 76 historical earthquakes, only those identified as mainshocks according to the declustered version of CPTI15 were considered to assess the exceedance area of the hazard maps. They are 71 in number and are mapped in Fig. 8b.

It is important to note that the considered dataset of historical earthquakes is far from complete as the CPTI15 counts more than 2200 mainshocks from 1117 to 1968. Therefore, no comparison with the expected fraction of territorial exceedance, as done in the previous section referring to a continuously monitored time interval, can be reasonably discussed. For these reasons, these 71 additional events were considered separately from those that occurred from 2008 to 2019. Nevertheless, this allows one to compute the fraction of the country that experienced at least one exceedance due to the considered historical events and enables further comparison of the hazard models considered.

### ShakeMap envelopes for instrumental earthquakes

In order to quantify the area in Italy subjected to least one exceedance in 12 years of earthquakes, ShakeMap envelopes for the earthquakes in Fig. 7b were developed^48^. This corresponds to taking, for each point of a grid, the largest ground motion intensity value among those provided by ShakeMap for the considered events. To do so, for each earthquake, ShakeMap data were preliminarily interpolated on a common grid featuring about 300,000 nodes discretizing Italy. (The points of this grid are also used as the sites to evaluate the fractional exceedance area of the country where it is estimated that at least one exceedance in 12 years has occurred.)

**Figure 9:**
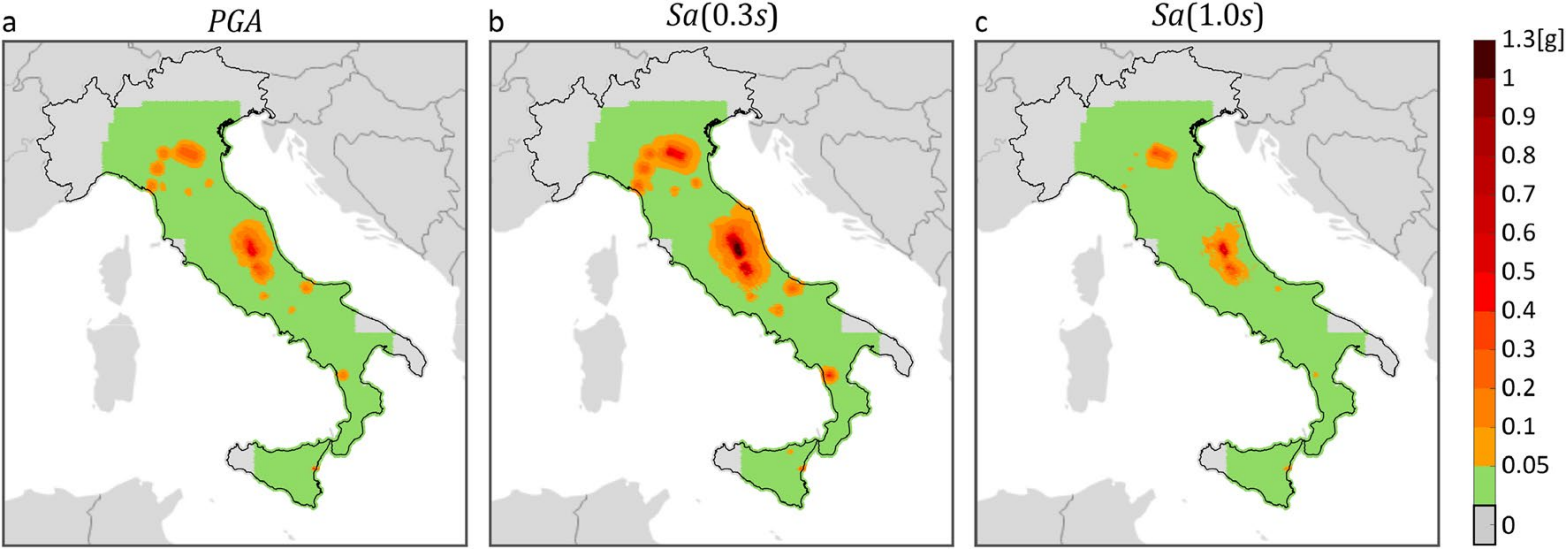
Largest ground motion intensities due to the M_w_ 4.45+ mainshocks that occurred in Italy from 2008 to 2019, in terms of $PGA$ (**a**), $Sa\left(0.3s\right)$ (**b**) and $Sa\left(1.0s\right)$ (**c**), according to ShakeMap envelopes. The maps were generated using the Matlab mapping toolbox version 4.10 (https://it.mathworks.com/products/mapping.html).

ShakeMap envelopes data are provided as supplementary material. Their graphical representation is given in Fig. 9, where panels a, b and c refer to $PGA$*,* $Sa\left(0.3s\right)$ and $Sa\left(1.0s\right)$, respectively. The figure is obtained by enveloping ShakeMap for mainshocks with M_w_ 4.45+, that is, the mapped ground motion intensities are used to quantify the exceedance area of MPS19 and ESHM20 hazard maps. The gray areas in each map include sites (i.e., the points of the grid) where ShakeMap intensity is zero or where there are no mainshock data available. The envelopes reveal that, for each $IM$, the largest intensity due to mainshocks that occurred between 2008 and 2019, is lower than 0.05 g in an area covering about 63% of the country. Comparatively large shaking values are found in central Italy, Emilia and a small area around Etna volcano in Sicily. For instance, the largest $PGA$, equal to 0.66 g, is due to the Viagrande (Sicily) 2018 earthquake (M_w_ 4.9); for $Sa\left(0.3s\right)$ and $Sa\left(1.0s\right)$, the largest value is equal to 1.32 g and 0.76 g, respectively, with the both of them being caused by the M_w_ 6.5 mainshock of the 2016–2017 central Italy seismic sequence.

### ShakeMap envelopes for historical earthquakes

**Figure 10:**
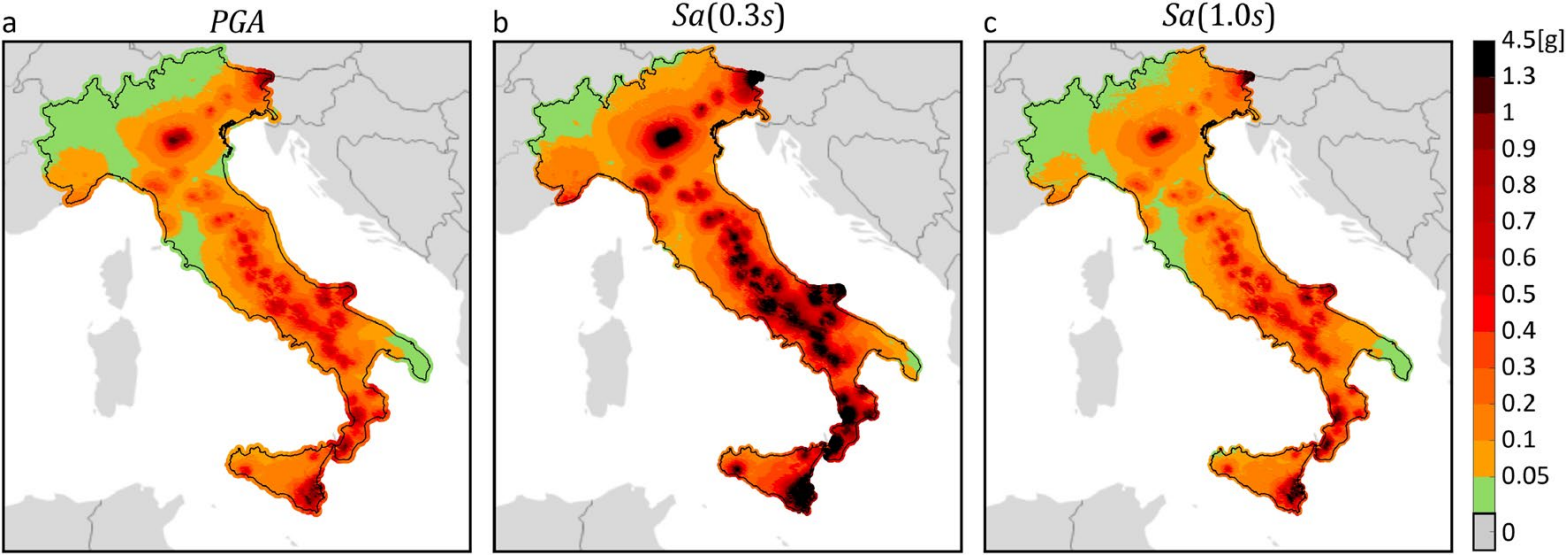
Largest ground motion intensities due to the historical M_w_ 6.0+ mainshocks, whose ShakeMap are available, occurred in Italy from 1117 to 1968, in terms of $PGA$ (**a**), $Sa\left(0.3s\right)$ (**b**) and $Sa\left(1.0s\right)$ (**c**), according to ShakeMap envelopes. The maps were generated using the Matlab mapping toolbox version 4.10 (https://it.mathworks.com/products/mapping.html).

The area in Italy subjected to least one exceedance due to historical mainshocks was quantified by comparing hazard maps with ShakeMap envelopes for earthquakes in Fig. 8b. Such envelopes were computed as discussed in the previous section and are provided as supplementary material. Figure 10, represents the envelope in terms of $PGA$*,* $Sa\left(0.3s\right)$ and $Sa\left(1.0s\right)$, from left to right. For each $IM$, the largest intensity due to mainshocks that occurred between 1117 and 1968 (the 71 in number whose ShakeMap is available, in fact), is lower than 0.05 g in an area which is significantly reduced with respect to what found considering mainshocks that occurred between 2008 and 2019, being equal to about 21%. In the case of $PGA$, the largest estimated value is larger than 1.0 g, and it is due to the M_w_ 7.3 earthquakes that occurred in 1693 near Siracusa (southeast Sicily). Looking at $Sa\left(0.3s\right)$, there is a not negligible fraction of the country, equal to about 5%, where the largest intensity estimated by ShakeMap is larger than 1.3 g, with the largest estimated shaking larger than 4.0 g, which is still due to the M_w_ 7.3 earthquake occurred in 1693 in Sicily. ShakeMap for this event also gives the largest estimated intensity across the country in terms of $Sa\left(1.0s\right)$, which is larger than 2.0 g.

### Quantifying exceedance areas

The comparison of the ShakeMap envelopes with the thresholds from seismic hazard maps enables quantifying the estimated fraction of the country that has been subjected to exceedance, at least once, due to mainshocks whose ShakeMap data has been considered in the envelopes themselves. Because ShakeMap accounts for soil site conditions, the ground motion intensities from the hazard maps are adjusted for the site conditions via amplification coefficients provided by the GMM considered in each PSHA study. For each hazard model and $IM$, the sites where the intensity from the envelope is larger than the threshold from the hazard map were counted for each return period. Dividing the number of sites where intensity from the envelope exceeds that from the hazard map by the total number of sites considered in the calculations (i.e., the number of grid points where the envelope was determined) gives the estimated fraction of the country subjected to at least one exceedance due to the mainshocks that envelope is built on.

**Figure 11:**
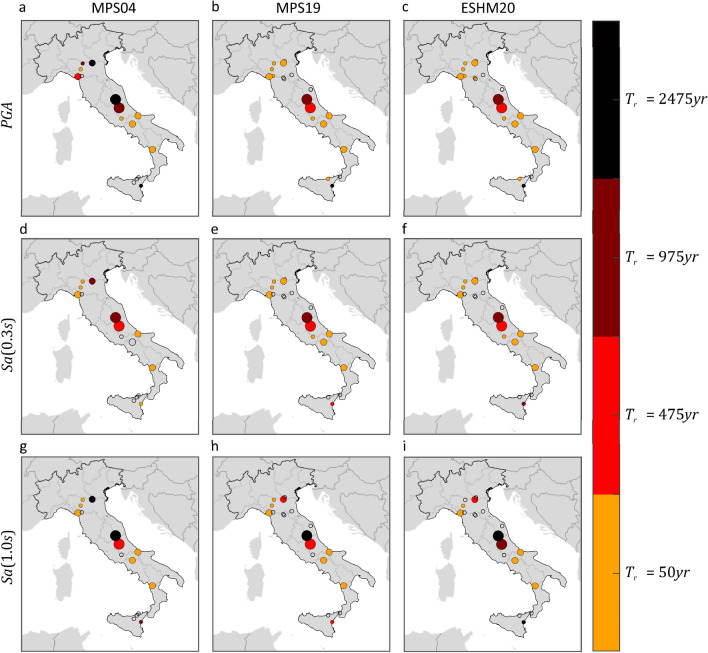
Mainshocks in Italy from 2008 to 2019 determining the largest $IM$ values and causing exceedance, at least at one site, of the PSHA intensity thresholds corresponding to three hazard models. The maps in the same column refer to the same PSHA model: (**a,d,g**) are from MPS04; (**b,e,h**) are from MPS19; (**c,f,i**) are from ESHM20. The maps in the same row refer to the same $IM$: (**a–c**) are $PGA$; (**d–f**) are $Sa\left(0.3s\right)$; (**g–i**) are $Sa\left(1.0s\right)$. The maps were generated using the Matlab mapping toolbox version 4.10 (https://it.mathworks.com/products/mapping.html).

**Figure 12:**
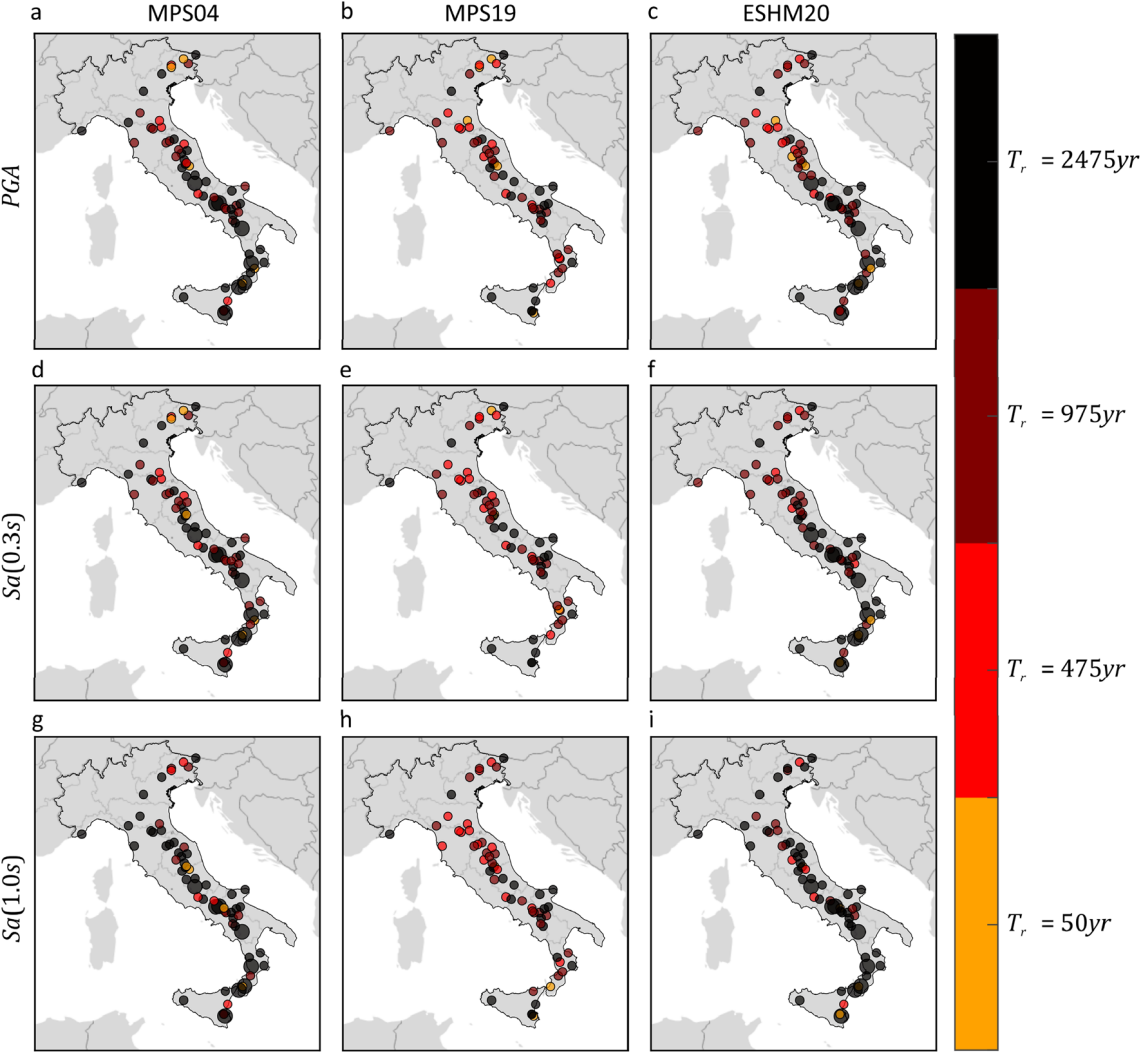
Mainshocks in Italy from 1117 to 1968, whose ShakeMap are available, determining the largest $IM$ values and causing exceedance, at least at one site, of the PSHA intensity thresholds corresponding to three hazard models. The maps in the same column refer to the same PSHA model: (**a,d,g**) are from MPS04; (**b,e,h**) are from MPS19; (**c,f,i**) are from ESHM20. The maps in the same row refer to the same $IM$: (**a–c**) are $PGA$; (**d–f**) are $Sa\left(0.3s\right)$; (**g–i**) are $Sa\left(1.0s\right)$. The maps were generated using the Matlab mapping toolbox version 4.10 (https://it.mathworks.com/products/mapping.html).

Figure 11 represents, for each $IM$ and hazard model, the mainshocks determining the intensity values provided by ShakeMap envelope for instrumental mainshocks. The figure also indicates if each earthquake causes exceedance, at least at one of the sites where it causes the intensity from the envelope, of the threshold from MPS04 (a–c), MPS19 (d–f) and ESHM20 (g–i) hazard maps. Gray markers depict mainshocks being causative of the largest intensity at some sites and yet not causing exceedance at any of them. The orange, red, amaranth, and black dots indicate earthquakes determining the largest intensity at some sites and also causing exceedance, at least at one of them, of the hazard thresholds with 50, 475, 975, and 2475 years return period, respectively. The figure shows that, for each $IM$ and hazard model, the mainshocks causing exceedance are reduced by more than half with respect to those causing the largest intensity. It can be also seen that, given the $IM$, the pattern of the maps seems to show a limited sensitivity to the PSHA model. In other words, for each $IM$, only a relatively few mainshocks contribute to the area where at least one exceedance of the hazard intensity has possibly occurred from 2008 to 2019, and the different PSHA models share several of them. Figure 12 represents the mainshocks determining the intensity values provided by ShakeMap envelope for historical mainshocks. It can be observed that, for each $IM$ and hazard model, each of the 71 mainshocks determines the intensity value provided by ShakeMap envelope and causes exceedance at least at one site.

### Supplementary Information

Supplementary Information 1.

Supplementary Information 2.

Supplementary Information 3.

## Data Availability

Hazard maps considered are given in Supplementary data1. ShakeMap are available at http://shakemap.ingv.it/shake4/. Shakemap envelopes for instrumental events are given in Supplementary data2. Supplementary data3 provides the envelopes for historical earthquakes. CPTI15 is available at https://emidius.mi.ingv.it/CPTI15-DBMI15/description_CPTI15.htm (last accessed March 2023). Declustered CPTI15 was provided by Dr. A. Rovida (INGV). ESHM20 data was provided by Dr. L. Danciu, who claims that they approximate the full EHSM20 logic tree.

## Code availability

PSHA was performed via REASSESS, which is available at http://wpage.unina.it/iuniervo/doc_en/REASSESS.htm.
